# Role of Diet and Nutrients in SARS-CoV-2 Infection: Incidence on Oxidative Stress, Inflammatory Status and Viral Production

**DOI:** 10.3390/nu14112194

**Published:** 2022-05-25

**Authors:** Fatiha Brahmi, Anne Vejux, Imen Ghzaiel, Mohamed Ksila, Amira Zarrouk, Taoufik Ghrairi, Soukena Essadek, Stéphane Mandard, Valerio Leoni, Giuseppe Poli, Dominique Vervandier-Fasseur, Omar Kharoubi, Adil El Midaoui, Atanas G. Atanasov, Smail Meziane, Norbert Latruffe, Boubker Nasser, Balkiss Bouhaouala-Zahar, Olfa Masmoudi-Kouki, Khodir Madani, Lila Boulekbache-Makhlouf, Gérard Lizard

**Affiliations:** 1 Laboratory Biomathématique, Biochimie, Biophysique et Scientométrie, Faculté des Sciences de la Nature et de la Vie, Université de Bejaia, Bejaia 06000, Algeria; khodir.madani@univ-bejaia.dz (K.M.); lila.makhlouf@univ-bejaia.dz (L.B.-M.); 2 Department of Biochemistry of the Peroxisome, Inflammation and Lipid Metabolism, University of Bourgogne Franche-Comte, 21000 Dijon, France; anne.vejux@u-bourgogne.fr (A.V.); imenghzaiel93@gmail.com (I.G.); mohamedksila44@gmail.com (M.K.); ess.soukaina@hotmail.fr (S.E.); nobert.latruffe@u-bourgogne.fr (N.L.); 3 Lab-NAFS ‘Nutrition-Functional Food & Vascular Health’, Faculty of Medicine, LR12ES05, University Monastir, Monastir 5000, Tunisia; zarroukamira@gmail.com; 4 Laboratory of Neurophysiology, Cellular Physiopathology and Valorisation of Biomolecules, (LR18ES03), Department of Biology, Faculty of Sciences, University Tunis El Manar, Tunis 2092, Tunisia; taoufik.ghrairi@fst.utm.tn (T.G.); olfa.masmoudi@fst.utm.tn (O.M.-K.); 5 Laboratory of Biochemistry, Faculty of Medicine, University of Sousse, Sousse 4000, Tunisia; 6 Laboratory Neuroscience and Biochemistry, University of Hassan 1st, Settat 26000, Morocco; boubker_nasser@hotmail.com; 7 Lipness Team and LipSTIC LabEx, UFR Sciences de Santé, INSERM/University of Bourgogne Franche-Comté LNC UMR1231, 21000 Dijon, France; stephane.mandard@u-bourgogne.fr; 8 Department of Laboratory Medicine, University of Milano-Bicocca, Azienda Socio Sanitaria Territoriale Brianza ASST-Brianza, Desio Hospital, Via Mazzini 1, 20833 Desio, Italy; valerio.leoni@unimib.it; 9 Department of Clinical and Biological Sciences, San Luigi Hospital, University of Turin, 10043 Orbassano (Turin), Italy; giuseppe.poli@unito.it; 10 Team OCS, Institute of Molecular Chemistry of University of Burgundy (ICMUB UMR CNRS 6302), University of Bourgogne Franche-Comté, 21000 Dijon, France; dominique.fasseur@u-bourgogne.fr; 11 Laboratory of Experimental Biotoxicology, Biodepollution and Phytoremediation, Faculty of Life and Natural Sciences, University Oran 1 ABB, Oran 31000, Algeria; omarkharoubi@gmail.com; 12 Department of Pharmacology and Physiology, Faculty of Medicine, University of Montreal, Montreal, QC H3C 3J7, Canada; adil.el.midaoui@umontreal.ca; 13 Faculty of Sciences and Techniques, Moulay Ismail University of Meknes, Errachidia 52000, Morocco; 14 Institute of Genetics and Animal Breeding of the Polish Academy of Sciences, Jastrzebiec, 05-552 Magdalenka, Poland; aatanasovmailbox@gmail.com; 15 Institut Européen des Antioxydants, 1b Rue Victor de Lespinats, 54230 Neuves-Maison, France; smeziane@ie-antioxydants.com; 16 Laboratory of Biomolecules, Venoms and Theranostic Applications, Pasteur Institute of Tunis, University of Tunis El Manar, Tunis 1002, Tunisia; balkiss.bouhaouala@pasteur.rns.tn; 17 Centre de Recherche en Technologie des Industries Agroalimentaires, Route de Targua Ouzemour, Bejaia 06000, Algeria

**Keywords:** COVID-19, medicinal plants, diet, nutrients, antiviral activity, antioxidant activity, anti-inflammatory effect

## Abstract

Coronavirus illness (COVID-19) is an infectious pathology generated by intense severe respiratory syndrome coronavirus 2 (SARS-CoV-2). This infectious disease has emerged in 2019. The COVID-19-associated pandemic has considerably affected the way of life and the economy in the world. It is consequently crucial to find solutions allowing remedying or alleviating the effects of this infectious disease. Natural products have been in perpetual application from immemorial time given that they are attested to be efficient towards several illnesses without major side effects. Various studies have shown that plant extracts or purified molecules have a promising inhibiting impact towards coronavirus. In addition, it is substantial to understand the characteristics, susceptibility and impact of diet on patients infected with COVID-19. In this review, we recapitulate the influence of extracts or pure molecules from medicinal plants on COVID-19. We approach the possibilities of plant treatment/co-treatment and feeding applied to COVID-19. We also show coronavirus susceptibility and complications associated with nutrient deficiencies and then discuss the major food groups efficient on COVID-19 pathogenesis. Then, we covered emerging technologies using plant-based SARS-CoV-2 vaccine. We conclude by giving nutrient and plants curative therapy recommendations which are of potential interest in the COVID-19 infection and could pave the way for pharmacological treatments or co-treatments of COVID-19.

## 1. Introduction

In December 2019, novel evolved coronavirus called COVID-19 appeared for the first time [1]. This local epidemic has converted to a global pandemic, and more than two years later the repercussions stay unclear and dreadful [2]. At the start of 2022, the COVID-19 pandemic has been accountable for more than 100 million stated contaminations and 5 million deaths with dramatic social consequences [2].

Examination of COVID-19 at the beginning of sickness and through to the progress of the infection differs from asymptomatic to acute pneumonia with severe respiratory distress syndrome, with the most usual symptoms being cough, shortness of breath, diarrhea, fever and tiredness [3,4]. Moreover, contaminated individuals by this RNA virus presented venous thromboembolic incidents linked to endothelial damage and hypercoagulability, immoderate inflammatory status with cytokine storm, immune dysregulation, cholesterol metabolism abnormalities, oxidative stress, hypertension and new onset diabetes [5,6,7,8].

The establishment of the vaccine has been the most substantial procedure to prevent acute COVID-19 and constitutes a key function in monitoring and reducing death. However, SARS-CoV-2 exerts its adaptative capabilities by mutation of its proteins. Thus, the vaccine becomes less efficient since only a unique modification of amino acid is capable to impact the viral replication, transmission or immune control avoidance.

The most recent variant, Omicron, includes more than thirty amino acid mutations in the spike protein and displays a higher transmissibility and evade capacity from vaccines [9].

In this context, it is important to identify natural and synthetic molecules capable of enhancing the host’s natural defenses and/or to counteract viral activity.

Up to now, medicinal and aromatic plants have been exploited in various aboriginal medical strategies along with ancestral medicines for treatment of sicknesses. So, a broad range of natural products can act as a subsidiary manual to freeing the several secrets regarding human pathologies [10]. Indeed, the evaluation of potential antiviral effect of different natural sources has acquired notable attention with the appearance and re-appearance of novel viruses and in view of the advancement of technologic resources that are accessible [11].

Research on new drug discovery from natural products can permit the identification of new lead molecules, which is progressed from a screening hit to a drug candidate through structural elucidation. Plants can lead to powerful molecules against SARS-CoV-2 as they have formerly demonstrated hopeful expectations for various pivotal situations generated by deadly pathogens [12]. 

Herbal medicines may offer efficacious medicines with various favorable impacts towards COVID-19 and these should be evaluated. Plant therapies may allow us to inactivate the virus, block its reproduction or spread or diminish the symptoms, thus not only decreasing suffering and declining mortality, but also diminishing further spread [13]. 

A range of natural products have been considered, and their effectiveness towards viral infections such as COVID-19 have been established. Several isolated natural molecules and different polar or apolar plant extracts such as *Artemisia annua*, *Agastache rugosa*, *Astragalus membranaceus*, *Cassia alata*, *Ecklonia cava*, *Gymnema sylvestre*, *Glycyrrhizae uralensis*, *Houttuynia cordata*, *Lindera aggregata*, *Lycoris radiata*, *Mollugo cerviana*, *Polygonum multiflorum*, *Pyrrosia lingua*, *Saposhnikoviae divaricate* and *Tinospora cordifolia* have demonstrated auspicious inhibiting impact towards coronavirus [4].

Meanwhile, important quantities of antiviral substances yielded from different plant species have been explored in several studies. Scientists around the world are testing therapeutic medication from available bioactive substances and they try to discover innovative molecules from herbal medicines towards COVID-19 [12]. 

In parallel, several works showed that nutritional status represents a crucial function in the immune system, supporting both innate and adaptive immunity and has a role in the control of inflammation [9]. Hence, human alimentation represents an elementary function in defending against contagious diseases. Persons with nutrient deficits have diminished blood cell generation which caused distressed defense cells and augmented hazard of contaminations. Diet assistance and food quality is tremendously critical to battle against and avoid contaminations, particularly in people infected by the COVID-19 [2]. So, some investigations indicate that the outcome of COVID-19 patients is linked with their nutritional status [9]. Indeed, natural molecules such as omega 3 and 9 fatty acids and tocopherols in large quantities in the Mediterranean diet have the ability to reduce 7-ketocholesterol (7KC) toxic effects [14,15,16] and could therefore greatly reduce the complications due to COVID-19 infection associated with increased levels of 7KC in the plasma of infected patients [5,6]. It has also been reported in some countries, that hospitalized or gravely sick young individuals are at a higher risk of malnutrition, and fast valuation and care of poor nutritious health can influence medical consequences [17]. As it links to the COVID-19 pandemic, an expected 5% of these ill persons need admittance to an intensive care unit. Dietary therapy should be among the essential elements of curative regimens [3]. Thus, discrepancies in dietetic lifestyles have been assumed as acting an important role in COVID-19 geographical and mortality rate variance [7]. 

It is also very important to point out that at the end of acute phase of SARS-CoV-2 pandemic (long COVID), a sequence of perpetual symptoms, which last more than 12 weeks from the commencement of the infection, can occur. Cognitive dysfunction and fatigue are principal symptoms joined by sleep perturbation, deficit of concentration, depression and pain. Changes in taste and smell, headache, dizziness, coordination troubles, memory loss, anxiety and insomnia are also discovered. Indeed, long COVID also affects several healthy young people who have not been hospitalized [18,19]. 

Therefore, during the COVID-19 pandemic, the secure uptake of natural products such as plants, micro- and/or macronutrients can be beneficial not only to reduce the infection but also to support the immune response during COVID-19, together with their effect on long COVID [9]. 

Nutrition may play a substantial function in influencing the susceptibility of long COVID. Nutrients, such as vitamins (B1, B6, B9, B12, C, D and E), fatty acids, minerals (iron, zinc) and oligoelements (selenium), are recognized to perform a significant role in protecting towards neuroinflammation and oxidative stress. They have a very positive impact on cognitive functions [20]. 

Additionally, secondary plant metabolites such as flavonoids inhibit neuroinflammation and reduce cognitive decline. Especially, luteolin (phenolic compound) is capable of crossing the blood–brain barrier and reducing both microglial and mast cell inflammation [9]. 

An extraordinary number of research studies have been launched to investigate preventative options and possible anti-COVID-19 therapies using natural products either from medicinal plants or different types of foods [21]. 

Accordingly, this review aims to understand the prospective functions of herbal medicine and the nutrients of various food groups with their antiviral, antioxidant and anti-inflammatory activities that can contribute to enhance immunity towards viral infections attributable to SARS-CoV-2. This can help medical doctors and allows citizens to make decisions based on natural medicine and suitable dietary choices in pandemic along with post-pandemic situations. 

## 2. Mechanism of SARS-CoV-2 Infection

With the aim of drawing up a therapeutical approach, it is necessary to understand the impact of COVID-19 on host targets. Several studies have been carried out to determine this effect [22,23]. In this review, it will help us to understand the mechanism of action of some natural products with regard to the infection of this virus. Most researches have adequately studied the mechanisms of COVID-19 entering host cells, and in specific the linkage of the spike (S) protein to its receptor, angiotensin-converting enzyme 2 (ACE2) and succeeding membrane fusion. The multistep SARS-CoV-2 entry process includes S protein synthesis and S protein structure conformational transitions necessary for association of the S protein with ACE2, engagement of the receptor-binding domain of the S protein with ACE2 receptor, proteolytic activation of the S protein by transmembrane protein serine 2 (TMPRRSS2), endocytosis and membrane fusion [24]. 

**Figure 1:**
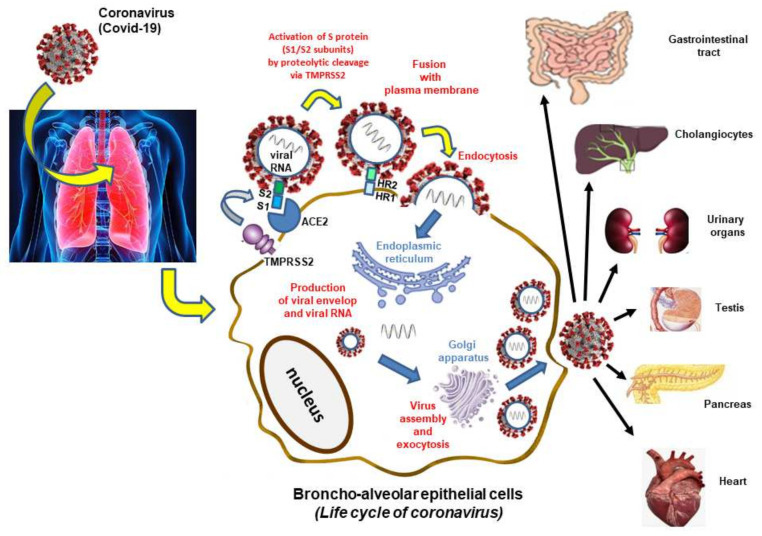
Mechanism of infection by SARS-CoV-2.

The spike (S) protein is made of two subunits (S1 and S2) and the partitioning and activation of the S protein are controlled by the intracellular TMPRSS2, also named furin to engender unlocked, fusion-catalyzed forms on the cell surface. This facilitates the earliest entrance of the virus. Cellular receptor ACE2 is used to enter the target cells. Notably, the heptad repeat 1 (HR1) and heptad repeat 2 (HR2) at the S2 subunit perform a few prominent functions in fusion regulating among the virus and host cell membrane. The HR1 and HR2 react to constitute a six-helix bundle, which allows the fusion of the two membranes. The organs which highly express ACE2 (cholangiocytes, small intestine and duodenum, urinary organs, testis, pancreas and heart) are vulnerable to SARS-CoV-2 infection [25] (Figure 1).

The SARS-CoV-2 targets a cellular receptor in the nasal cavity, where it would multiply before disseminating in particular in the lungs. The main stages of human cell infection and virus replication take place at the plasma membrane level, in the cytoplasm (endoplasmic reticulum, Golgi apparatus) and are successively as follows: (1) Activation of the viral protein spike by furin and attachment to the ACE2 receptor by the S1 subunit which recognizes and binds to this cellular receptor. Beyond attaching the virus to the cell, the role of the spike protein is to induce fusion between the viral envelope and a cell membrane. It is a heptad repeat 1 (HR1) and heptad repeat 2 (HR2) that constitute a six-helix bundle, permitting the two membranes to fuse. This step, which corresponds to endocytosis, requires the spike protein to be cut again by a protease called TMPRSS2 (transmembrane serine protease 2). (2) Synthesis of viral messenger RNA and duplication of viral genomic RNA: when the virus has entered the cell, it releases its genomic RNA. The virus RNA polymerase synthesizes messenger RNA and copies of genomic RNA that will be used to form new virus particles. (3) Multiplication of viral particles by exploiting the cellular machinery: the virus messenger RNA uses the cellular machinery to synthesize the viral polyproteins it encodes. Once the polyprotein has been synthesized, a viral protease cuts it and allows the formation of functional viral particles. (4) The viral nucleocapsid is gathered from genomic RNA and N proteins in the cytoplasm, and thereafter bud into the lumen of endoplasmic reticulum (ER)-Golgi intermediate cavity. Virus particles are then liberated from infected cells by exocytosis. The receptor is present not only on the cells of the nose and the lungs but also in the digestive system, the heart and to a lesser extent in the kidneys and the liver. SARS-Cov-2 can therefore infect all these organs.

Both ACE2 and TMPRSS2 mRNA have been reported in different tissues. The penetration of the virus into the cells and fixation on the ACE2 receptor can generate damage including thrombosis and hypoxia immune response against the virus and cytokine storm [26]. 

Other receptors can manage SARS-CoV-2 infection such as dendritic cell-specific intercellular adhesion molecule-3-grabbing nonintegrin (DC-SIGN; genotypes CD209), liver/lymph node-specific intercellular adhesion molecule-3-grabbing integrin (L-SIGN; CLEC4M) and CD147 which explicate its elevated infectiveness [27].

TMPRSS2 inhibitors could constitute an interesting target for the prevention or treatment of COVID-19 infection [25,26]. Furthermore, different proteins or genes of SARS-Cov-2, such as the main protease, have been targeted to screen medicines; however, no small molecules have been conclusively shown to be able to treat COVID-19 patients [28]. Thus, de Oliveira et al. [29] in their in silico study indicated potential challengers from herbal compounds to inhibit the SARS-CoV-2 main protease (Mpro). They discovered that non-polar and polar groups with the occurrence of hydrogen bond acceptors have a substantial function in the herbal compounds–Mpro interactions.

## 3. Medicinal Plants and Their Metabolites Used in Case of COVID-19

### 3.1. Medicinal Plants and Their Extracts

Around 35% of the worldwide medicine market has been exchanged by medicinal products formulated by natural herbs. Hence, plants could be an additional source of molecules in the therapy towards COVID-19 [12].

Supportive therapies are used to regulate additional difficulties and organ damage generated by COVD-19. Moreover, the implementation of both modern and traditional therapies might diminish the severity of the disease and symptoms, death rate and side effects [11]. 

The specified mechanisms of contemporary medications under examination for COVID-19 therapy comprise the inhibition of fusion of SARS-CoV-2 with human cells, a decline in endosomal acidity, cell-membrane-generated vesicles for transport of the virus inside the host cell where the virus can replicate and obstruction of the formation of pro-inflammatory cytokines [30]. Moreover, major domains of novel medication targets are RNA-dependent RNA polymerase of the virus, cell membrane receptors, and spike proteins [4].

Plant bioactive substances can also inhibit (i) the enzymes implicated in the replication cycle of CoVs such as papain-like protease and 3CL protease, (ii) the fusion of the S protein of coronaviruses and ACE2 of the host and (iii) the associated cellular signaling pathways [12].

Natural products can probably stimulate either one or a combination of the cited impacts and the antiviral process of plant extracts depends on the structure and replication mechanism of the viruses. On the other hand, some herbs aid the enhancement of the natural antiviral immunity of the organism. In line with this, medicinal herbs can be a substitute for synthetic drugs especially since plants are the elementary origin of medical care for almost 85% of the world inhabitants and more than 40% of medications found in pharmacies originate from vegetables and microbial-based natural products [4].

Plants and natural products were exploited by populations to avoid COVID-19 since they are accessible as a first line of protection with a view to stimulate immunity and hence make the body more resistive to contaminations. They can also intervene in hygiene measures by purifying the air. A range of bioactive components are found in these herbs and many herbs are recognized for their antioxidant, anti-inflammatory and even antiviral effects [31].

COVID-19 symptoms create inflammation and hemotoxicity, which means that blood-purifying plants with anti-inflammatory, antioxidant and antiviral activities could be perceived as potential cures for COVID-19 infections [32].

Consequently, numerous research studies focused on the screening and characterization of prospective anti-inflammatory, antioxidant and anti-antiviral medicinal plants in the pandemic context. So, in this section, we will focus on plants that have these specific properties. 

There are several surveys identifying the employment of diverse medicinal plants in ethnomedicine during the COVID-19 pandemic across various regions worldwide, particularly in Asian countries (China, India, Japan and Pakistan) and Africa (Algeria, Cameroon, Ethiopia, Morocco, Nigeria). However, we will only focus on those that present important results and evidence. 

In fact, many researchers have pointed out the effects of the chosen medicinal plants on this virus. Most of the studies have been focused on Chinese plants, and it has been shown that traditional Chinese medicines (TCMs) decreased certain symptoms of COVID-19, such as fevers and reduced the viral load considerably [33]. 

For instance, *Glycyrrhiza glabra* (liccorice) was considered potential therapy for COVID-19 because of its prior effectiveness towards the SARS epidemic of 2003. In addition, different Chinese investigations examined chosen TCMs towards COVID-19 and stated that some of them, especially *G. glabra*, diminished the symptoms of this pandemic disease, notably fevers and reduced the viral load significantly [13]. 

*Terminalia ferdinandiana* Exell (commonly known as Kakadu plum) has good potential in decreasing the symptoms of COVID-19, thereby saving lives. The extract of this plant possesses exceedingly elevated antioxidant amounts such as vitamin C (~900 times (g/g) the ascorbic acid amount of blueberries) which boosts the immune system. High ascorbic acid levels enhance the immune system, thereby reducing the likeliness of infection by SARS-CoV-2 [13].

Noticeably, many research studies have demonstrated that Kakadu plum extracts inhibit the release of pro-inflammatory cytokines and promote the liberation of anti-inflammatory cytokines [13].

The Malagasy Institute for Applied Research formulated an herbal tea based on *Artemisia annua*, alleging prophylactic and therapeutic effects towards COVID-19 [34]. The effect of the cited herb towards SARS is recorded and has been approved in COVID-19 therapeutic practices [35]. 

*Sambucus javanica* subsp. chinensis (Lindl.) Fukuoka (Chinese elder) extract also exercised auspicious anti-human coronavirus properties. This extract significantly reduced virus yield, plaque generation and virus linking [36]. Moreover, there is preclinical indication that *Sambucus nigra* L. (elderberry) prevents the replication and viral linking of the human coronavirus NL63. This plant is the most efficient in preventing or fighting coronavirus infections at the early stages [37]. 

*Rhodiola rosea* L. (Golden root) has great immunoregulatory action and attenuates inflammatory harm since it regulates the differentiation of immune cells, activates inflammatory signaling pathways and secretes inflammatory factors [38]. *Andrographis paniculata* (Bitter weed) inhibits the augmented NOD-like receptor protein 3 (NLRP3), caspase-1 and interleukin-1β (IL-1β) particles which are strongly implicated in SARS-CoV [39]. 

Among the other reported species, the decoction of *Qingfei Paidu*, *Gancaoganjiang*, *Sheganmahuang* and of *Qingfei Touxie Fuzheng* exhibited promising effects [33].

Professional persons from the Zhongnan Hospital of Wuhan University comprised the usage of conventional remedies in the guidance for the treatment and prevention of COVID-19. Furthermore, to cure the infection, the specialists advise the utilization of several plant combinations depending on the pathology phase [40]. Six species are used in the TCM Yupingfeng powder, which are as follows: *Astragalus mongholicus* Bunge, *Glycyrrhiza glabra* L., *Saposhnikovia divaricata* (Turcz. ex Ledeb.) Schischk., *Atractylodes lancea* (Thunb.) DC., *Atractylodes macrocephala* Koidz., *Lonicera japonica* Thunb. and *Forsythia suspensa* (Thunb.) Vahl. [41]. 

Likewise, numerous TCM prescriptions were formulated including the lung cleansing and detoxifying decoction (a mix of 21 plants and natural products) which is clinically assessed. This formula was efficient in 90% of the 214 enrolled COVID-19 patients and the treatment of 1262 patients, including 57 with severe symptoms, revealing that 99.28% of the patients were cured and none with mild symptoms showed severe symptoms. So, this preparation has been extensively adopted in 28 regions and has aided to the comparatively bass death proportion in between COVID-19 patients in China [42]. 

In India, Triyaq-e-Araba is also a formulation employed as a detoxifying and a potent antiviral agent. It includes *Laurus nobilis* L. berries, *Bergenia ciliate* (Haw.) Sternb. stem, *Aristolochia indica* L. roots and *Commiphora myrrha* (Nees) Engl [11]. 

In Ayurveda (an oriental holistic and preventive medicine), *Tinospora cordifolia* (Guduchi) and *Emblica officinalis* (Indian gooseberry) have immunity-boosting properties. While *Phyllanthus* spp., *Andrographis paniculata* (Creat), *Glycyrrhiza glabra* (Licorice), *Withania somnifera* (Ashwagandha) and *Curcuma longa* (Turmeric) have antiviral effects [43]. In metaanalysis research and in line with docking studies, it was revealed that *C. longa* can be employed as a preventive towards COVID-19 [32].

*Tinospora cordifolia* (Giloe), *Ocimum tenuiflorum* (Tulsi), *Emblica officinalis* (Indian gooseberry) and *Linum usitatissimum* (Linn.) (Flax seeds) have been traditionally employed as herbal remediations for various ailments since the early ages in India. They have demonstrated strong immunomodulatory, antioxidant and anti-infective activities [11]. 

Aqueous extract of *Withania somnifera* (Ashwagandha) inhibits SARS-CoV-2 from entering to cells by preventing liaisons between the viral S-protein receptor connecting domain and host ACE2 receptor [44]. 

AYUSH systems of medicine propagate general preventive measures aimed at preventing the spread of infection such as sustained general health by dietary modifications and herbal drugs. So, numerous formulations were developed including AYUSH kwath which is a mixture of herbs that boost immunity and are active remedies to various viral diseases, it includes *Ocimum sanctum* L. (Holy basil) leaves, *Piper nigrum* L. fruits (Black pepper), *Cinnamomum verum* J. Presl. stem barks (Cinnamon) and *Zingiber officinale* Roscoe rhizomes (Ginger) [11]. 

*C. verum*, which displays low toxicity, and rhizome of *Z. officinale* are employed to relieve fever and other COVID-19 symptoms in Africa [45,46]. It is worth pointing out that several other studies have demonstrated the antiviral activity of other plants which can be used against COVID-19 including *Rosmarinus officinalis* Linn. (Rosemary) in Africa [45,46]. 

Interestingly, in Morocco, based on an ethnobotanic study, 67.04% of interviewees employed medicinal plants to boost their immunity, disinfect the air or treat respiratory tract infections that may be linked to coronavirus. The most mentioned species that strengthen the immunity system were *Olea europaea* L. (Common olive), *Vitis vinifera* L. (Common grape) and *Allium sativum* L. (Garlic) [31]. This last one can be considered a promising plant that can act against COVID-19 since it exposed an inhibitory activity on viral replication [47]. Its extract prevents influenza A virus by inhibiting the synthesis of viral nucleoproteins and polymerase activity [48]. However, *Eucalyptus globulus* (Blue gum), *Trigonella foenumgraecum* (Fenugreek) and *Aloysia triphylla* (Lemon Verbena) were the species employed to alleviate some respiratory infection symptoms [31]. Additionally, Flouchi and Fikeri-Benbrahim [49] showed the efficiency of *Cinchona officinalis* Linn. (Quinquina), and *Thymus vulgaris* (Thyme) in diminishing and avoiding the hazard of contamination and in curing some COVID-19 symptoms. 

Among the group of plant prevention agents during the coronavirus pandemic, we can also cite *Nigella sativa* L.’s (Black cumin) bioactive constituents that have been noticed as promising inhibitors of COVID-19 in molecular docking work [50]. 

### 3.2. Major Plant Metabolites

According to Bhuiyan et al. [12], plant metabolites represent a potential anti-SARS-CoV-2 effect; therefore, it is interesting to study molecules for the increased optimization and generation of medical procedures to fight COVID-19 and also subsequent pandemics that are generated by viruses. These authors suggested that about 76 natural metabolites from plant species can be successfully active towards COVID-19.

#### 3.2.1. Polyphenols

Polyphenols (or phytophenols) are ubiquitous compounds produced by all plant species [51]. Besides their antioxidant properties, they have various physiological aspects: cell signaling, interactions with the environment (color, flavor), metal chelation, and defense against aggression either biotic (virus, bacteria, fungi infection) or abiotic (radiation, ozone, dryness) or sensor for pesticides. Several studies also support that they can stimulate immune response in cancer [52]. 

In animals, including humans, polyphenols have strong antioxidant effects on metabolic homeostasis. They prevent lipid, protein and nucleic acid oxidation. They also modulate the immune system by decreasing low grade inflammation. Polyphenols are good antiseptics and prevent some viral infection such as influenza virus, human immunodeficiency virus (HIV) and also SARS-CoV-2 [53]. 

A large variety of antiviral substances were revealed in 219 medicinal plants from 83 plant families, pre-eminent are polyphenols. Some polyphenol compounds (30-(3-methylbut-2-enyl)-30, 4-hydroxyisolonchocarpin, broussochalcone A, 4,7-trihydroxyflavane, broussochalcone B, papyriflavonol A, kazinol A, kazinol B, kazinol F, kazinol J and broussoflavan A isolated from *Broussonetia papyrifera* demonstrated auspicious effects towards SARS-CoV. Particularly, papyriflavonol A recorded an excellent effect against SARS-CoV (IC_50_ 3.7, l M) [12]. 

Therapy with some flavonoids (hesperidin and quercetin), administered with indomethacin as a non-steroidal anti-inflammatory medicine with antiviral activities and with a low dose of aspirin as an anti-aggregating property, was suggested to be successful after 3 days from the initiation of the symptoms of SARS-CoV-2. This treatment displayed a decrease in the severances of COVID-19 and a decrease in the percentage of hospitalizations [9]. 

Furthermore, SARS-COV-2 contamination triggers an immune response and an oxidative response, especially to the lungs. Liu et al. [54] have shown that interleukin (IL)-6 is one of the leading factors causing death in COVID-19 patients through cytokine release. Recently, Pincemail et al. [55] reported an oxidative stress increase in COVID-19 patients hospitalized in an intensive care unit for severe pneumonia. Trujillo-Mayol et al. [56] recalled the importance of antioxidant and vitamin D intakes during the SARS-CoV-2/COVID-19 pandemic. Therefore, vulnerable populations such as the elderly and obese individuals should benefit from antioxidants and vitamins especially through the Mediterranean diet to improve their antioxidant response. Although evidence remains scarce, there is some indication that a healthy diet, along with supplemental antioxidant intake, is beneficial to COVID-19 patients.

Since the start of the COVID-19 pandemic, some hypothesis merged about a possible preventative effect of polyphenols towards SARS-COV-2 effects. Moreover, many reviews and present papers demonstrate some interesting data. So far, the prevailing hypothesis is the following: SARS-CoV-2 replication can be inhibited by polyphenols which are derived from edible plants [28]. 

The in vitro effects of polyphenols on COVID-19 were demonstrated by their binding on the main protease involved in the virus replication and enzyme inhibition. The authors have screened a series of plant flavan-3-ols and of pro-anthocyanidins for instance: (+)-catechin (CA), (-)-epigallocatechin (EGC), (+)-gallocatechin (GC), (-)-epiafzelechin (EAF), (+)-afzelechin (AF), (-)-epicatechin-3-O-gallate (ECG), (+)-catechin-3-O-gallate (CAG), (-)-gallatechin-3-O-gallate (GCG) and (-)-epigallocatechin-3-O-gallate (EGCG). On the other hand, they tested plant extracts rich in such polyphenols. Flavan-3-ols are present in fruits, drinks (grape extracts), parsley, strawberries, blackcurrants, blackberries, cassis, cocoa, chocolate and green tea. 

The dimers of pro-anthocyanidins A1, A2, B1 and B2 are powerful antioxidants with the following properties: antiviral, antibacterial, anticancer, anti-vascular alterations and anti-aging activities.

Two approaches have been undertaken: (1) Modeling (docking) of these molecules to protease Mpro and (2) measurement of the protease Mpro activity. According to Figure 1, the main protease is involved in maturation of viral protein precursors synthesized by cell protein synthesis machinery oriented for the benefits to viral multiplication.

Six compounds bind in three of the four sites S1, S’1, S2, S4 located in the binding pocket of the peptide N3 inhibitor of Mpro. The highest affinity scores are for EAF, AF and CA, and the weakest for PA2 and PB2.

IC_50_ inhibition averages of measured protease activities are: 3 μM (CAG), 5 μM (ECG), 6 μM (GCG), 7 μM (EGCG) and 75 μM PB_2_. The other compounds do not show any inhibitory activities.

Furthermore, assays have been performed on extracts rich in CAG, ECG, GAG, EGCG and PB2 where results of IC_50_ are the following: green tea (3 µg/mL), grape muscadine (30 µg/mL), cocoa (153 µg/mL) and dark chocolate (256 µg/mL).

In conclusion, (1) the inhibitors’ effects are closely linked to affinity on the protease and (2) galloylation and these natural compounds are not immunizing drugs but can be preventive nutrients such cocoa, grape and green tea. These results have recently been confirmed by Bahun et al. [57].

In silico study shows that polyphenols can inhibit SARS-CoV-2 Mpro and RdRp efficiently and flavonoids exhibited powerful antiviral effects towards SARS-CoV. For example, apigenin and quercetin displayed effects towards SARS-CoV by inhibiting Mpro enzymes with an IC_50_ of 38.4 μM and 23.8 μM, respectively [12]. 

In addition, resveratrol (a polyphenol of the stilbene chemical family), under its trans-configuration, is a peculiar promising polyphenol for COVID-19 infection prevention. Indeed, resveratrol is produced in huge amounts by grape plants in response to fungi infection and accumulates in grape berries, especially in the skin. Resveratrol is present in red wine in significant amounts [58,59]. Resveratrol is a strong antioxidant [60] and shows natural anti-inflammatory properties. Its preventing properties have been verified towards the COVID-19 infection. For instance, resveratrol [61] and its analogue hopeaphenol [62], *Polygonum cuspidatum* extracts [63] and pterostilbene [64] inhibit SARS-CoV-2 replication. It has been proposed [65] that resveratrol may switch off interference between a link between COVID-19 and obesity via the inhibition of HIF-1α to HIF-2α (Hypoxia-inducible factor).

McLachlan [66] and Perrella et al. [67] have studied the activation of ACE2 receptor by resveratrol and other natural compounds. In an empirical investigation, it has proposed that an association of resveratrol and copper at 5.6 mg and 560 ng, respectively, orally once every 6 h has substantially reduced the death rate of very sick COVID-19 patients [68]. Kelleni [69] has proposed nanocarriers, resveratrol/pterostilbene-zinc nanoparticle administration to COVID-19 patients as adjuvant therapy. The anti-inflammatory and immunomodulatory activities of the associations were well known and can be considered as a main pharmacokinetic benefit for COVID-19. 

#### 3.2.2. Terpenoids

Terpenoids or isoprenoids constitute the major class of secondary metabolites only containing carbon, hydrogen and oxygen atoms [70,71]. Several terpenoids act as defensive constituents towards microorganisms. So, multitude terpenoids have marked pharmacological effects and are intriguing for medicine and biotechnology. Around 36,000 specific structures of this class have been mentioned [72].

In humans, terpenes are known to have anti-inflammatory, analgesic, antiviral and antibacterial activities. Regarding antiviral properties, studies have shown potential efficacy against human immunodeficiency virus 1, bronchitis virus, herpes simplex virus and West Nile virus [73,74,75,76].

Ten diterpenes, two sesquiterpenes and two triterpenes demonstrated an anti-SARS effect with IC_50_ of 3–10 μM. In in silico work, it has been shown that terpene Ginkgolide A can strongly inhibit the SARS CoV-2 protease enzyme [12]. In addition, Glycyrrhizin, a triterpene found in licorice roots, could inhibit SARS-CoV replication in vitro [77] and its efficacy was also obtained in patients [78]. Squalene, a natural triterpene which is a precursor of sterols and other bioactive terpenoids, was extracted from pumpkin seed oil. Then, it was assessed in the form of a nanostructure in a clinical study to treat COVID-19. It showed marked properties by reducing fever and cough during the treatment period and the treated patients did not need oxygen therapy [79].

Another study showed that *Laurus nobilis* L. essential oil, containing β-ocimene, 1,8-cineole, α-pinene and β-pinene, has antiviral activity against SARS-CoV [80]. An NT-VRL-1 formulation, containing about 30 terpenes with β-caryophyllene, eucalyptol and citral as main constituents, was tested on human lung fibroblast cells MRC-5 [81]. This formulation has antiviral effects which are amplified when used with cannabidiol. These effects would be most effective when the formulation is used prior to exposure to the virus, the compounds would affect the attachment and entry of the virus into cells [81]. An in silico study by Ibrahim et al. [82] focused on a protease involved in viral replication, blocking the activity of this enzyme would block viral replication and thus SARS-CoV-2. They screened in silico via their binding affinity to the protease involved in SARS-CoV-2 replication, biologically active terpene metabolites identified from a coral reef community unique to the Red Sea by comparing them to lopinavir (a known inhibitor of this protease, proposed as a treatment for COVID-19). Through molecular docking calculations, molecular dynamics (MD) simulations and molecular mechanics/generalized born surface area binding energy calculations, they identified a candidate molecule—erylosides B (226) [82]. A review written by Meeran et al. [83] evaluates the hypothesis that limonene, a cyclic monoterpene that constitutes about 98% of the essential oils in the peel, leaf and flower of many citrus fruits, including oranges, mandarins, lemons, pummelos, grapefruits and limes, could be used in the fight against COVID-19 due to its immunomodulatory, anti-inflammatory and antiviral properties. An interesting diagram summarizes the proposed possible effects of limonene and its possible involvement in SARS-CoV-2 infection. 

In the following section, we will pay more attention to carotenoids and phytosterols.

##### Carotenoids

Carotenoids are the most abundant plant pigments responsible for the red, orange, yellow and green colors of fruits, vegetables, flowers and algae, with 700 different fat-soluble compounds identified. They can be easily extracted in ether petroleum. They are not synthesized by humans, and there are only 50 compounds in food that can be absorbed and metabolized [84]. Some carotenoids are in the majority, such as β-carotene, β-cryptoxanthin, α-carotene, lycopene, lutein and zeaxanthin, they account for 95% of the carotenoids present in the blood. This family is divided into two groups: xanthophylls (containing oxygen, hydroxyl, epoxy, carboxyl group for example): lutein, zeaxanthin and β-cryptoxanthin; and carotenes (hydrocarbons not containing oxygen): β-carotene, lycopene and α-carotene. Carotenoids are described as having antioxidant properties [85] and as being able to interact with certain cellular signaling pathways involved in the suppression of oxidative stress and inflammatory processes [86]. The review by Khalil et al. presents the chemical composition, classification, natural sources and biopharmaceutical activities of a number of carotenoids and may provide useful information for their use [87]. One team studied the antiviral activity of two marine carotenoids against SARS-CoV-2 virus entry: fucoxanthin, a polar, orange xanthophyll, found in several brown algae, and siphona xanthin, a polar xanthophyll, found in green algae and an oxidative metabolite of lutein [88]. Molecular docking studies showed that siphona xanthin could bind to the ACE2 binding region of the SARS-CoV-2 spike protein. These data were confirmed by in vitro experiments on HEK/ACE2 cells infected with a SARS-CoV-2 pseudovirus. In the review by Khalil et al. [87], the authors analyzed the existing bibliography and gathered all the existing data concerning the impact of carotenoids (β-carotene, α-carotene, β-cryptocanthin, lutein/zeaxanthin, lycopene, astaxanthin, crocins and crocetin (present in high amount in saffron) [89], phytoene and phytofluene and fucoxanthin) on inflammation, immunity and immune function, as well as on their antiviral activity. As a result of this analysis, the authors suggest that carotenoids could be used as potential drugs (with the necessary restrictions associated with certain supplementations) to combat the inflammatory storm resulting from COVID-19 infection, but also to boost the immune response (effects on several anti-inflammatory and antioxidant pathways) [87]. The authors targeted an interesting candidate for future development: astaxanthin, because of its innumerable properties and antioxidant activities against several symptoms of viral infections, such as inflammation and its acceptable safety profile. It is closely followed by lycopene, crocin and crocetin.

##### Phytosterols

Currently, several studies have shown that cholesterol oxidation products, called oxysterols, may have antiviral activities [90] and may be involved in the COVID-19 infection which can lead to a severe acute respiratory syndrome (SARS-CoV-2) that can be fatal especially in the elderly and obese subjects. This is the case for 27-hydroxycholesterol (27-OHC) and 7KC, for which a decrease and an increase in plasma levels are, respectively, observed in elderly subjects with mild and severe forms of the COVID-19 infection [5,6]. Meanwhile, Foo et al. [91] demonstrated in their review study that oxysterols including 7KC can be deleterious in acute viral infections. A diseased function of 7KC has been advocated with various viral pathogens because of its recognized cytotoxic activities at elevated amounts. 7KC stimulates a pro-inflammatory phenotype in macrophages and can with other oxysterols participate in immoderate inflammation. Furthermore, antiviral activities against COVID-19 have been described with synthetic oxysterols [92]. Withaferin A (WFA), a steroidal lactone with anti-inflammatory and anti-tumorigenic properties, also binds to the SARS-CoV-2 viral spike protein [93]. In addition, among 117 steroidal plant-derived pregnanes (PDPs) which were docked in the active regions of human glucocorticoid receptors (hGRs) in a comparative molecular docking analysis, 20 were selected and analyzed for their possible interactions with the human Janus kinases 1 and interleukins-6 and SARS-CoV-2 3-chymotrypsin-like protease which is a papain-like protease and RNA-dependent RNA polymerase [94]. The most efficient PDPs were bregenin, hirundigenin, anhydroholantogenin, atratogenin A, atratogenin B, glaucogenin A, glaucogenin C and glaucogenin D [95]. Altogether, these data suggest that plant sterols called phytosterols (cholesterol derivatives with either an extra methyl or ethyl group on carbon 24 or an extra double bond on the side chain [96,97]) may have an impact on the COVID-19 infection. To counteract this type of infection, traditional medicines using medicinal plants containing phytosterols are therefore a rational solution. Among these traditional medicines, Chinese medicine is an important source of substances potentially rich in phytosterols (in particular β-sitosterol) capable of acting against the viral infection and/or its consequences at the level of the immune, bronchial, epithelial and vascular endothelial cells by intervening in the MAPK, NF-κB and TLR signaling pathways on the basis of in silico analyses [98]. A molecular docking strategy targeting the Mpro protein (3CL protease from coronavirus SARS-CoV-2) involved in viral replication constitutes a reliable strategy to identify drugs with antiviral activities which could be used in infected patients [99]. In this context, phytosterols from an Ayurvedic plant, *Boswellia serrata*, are believed to have antiviral activities against COVID-19 [100]. From an in silico study, using the admetSAR tool along with the SwissADME and Molinspiration chemoinformatics tools, it was found that castasterone, which is a brassicasterol, could also be efficient against the SARS-CoV-2 Mpro [101]. Furthermore, due to the ability of phytosterols, such as β-sitosterol, to interact with the plasma membrane, including lipid rafts, these molecules could also oppose viral infection by inhibiting the endocytosis that promotes intracellular virus accumulation [102]. Based on the small number of studies carried out either in silico or in vitro, it appears however that phytosterols can have an effect on both the viral infection (virus entry into the cells, viral cycle) and also on the inflammatory process, which makes these molecules significant in the fight against the COVID-19 infection in infected patients. 

Regularly studied medicinal plants and their active metabolites which are recommended to be used against the COVID-19 infection are summarized in Table 1.

## 4. Major Food Groups Efficient in COVID-19 Pathogenesis

Some nutrients such as vitamins A, B6, B12, C, D, E, folates and trace elements, (zinc, iron, copper, selenium, and magnesium) are formerly recognized as immune system enhancers. Deficits in these molecules unfavorably impact the activation of the immune system in infections. Different searches advised the earliest usage of molecules including zinc, selenium and vitamin D, together with other micronutrients, to increase resistance to COVID-19 [9].

Food has evolved into a substantial element in the treatment of persons during COVID-19. A diminution in food consumption during hospitalization is immediately related to deterioration clinical consequences [2].

It has been stated that diet complementation with some nutraceuticals performs a basic function in the treatment of respiratory symptoms, since several foods and their derivatives offer an immune response to respiratory viruses. Moreover, they regulate the activity of the inflammation generated by COVID-19 [113].

Suitable nutrition is necessary for immune system cells to play their role perfectly. Hence, this allows immune cells to begin efficient reactions towards germs, solve the response quickly and avert any underlying chronic inflammation [114].

During this pathogenesis it is essential to take care of nutritional practices, taking a healthy and balanced nutritional plan with high quantities of minerals, vitamins and antioxidants. Micronutrients can boost immune function too [115].

Dietary characteristics including unavailability of nutrient-rich food, changes in dietary pattern and more prone to eat processed food, malabsorption and maldigestion of food and excessive alcohol consumption compromised the immune system which increased the risk of infection [1].

### 4.1. Macronutrients

In the immune system, the alimentary elements that induce malfunctioning are the deficient consumption of macronutrients and source of energy. Some macronutrients possess special functions in developing and preserving an efficient immune system in diminishing chronic inflammation [114]. Protein hydrolysates boosted barrier function and IgA generation in animal models [116]. The amino acid arginine is vital for the formation of nitric oxide by macrophages [114].

Glutamine offers a substantial energy resource for cells implicated in immune responses. Furthermore, it helps in the nucleotide synthesis, notably appropriate for speedily dividing immune cells over an immune response. In the case of infection, the content of glutamine intake by immune cells is equal or higher than that for glucose. Glutamine intervenes in the roles of neutrophils, macrophages and lymphocytes [117].

COVID-19 ill patients receiving an enteral nutrition for >7 days and getting a considerable daily level of proteins and calories per kilogram showed a bass death rate comparable with patients with a less than suitable protein provision [2].

Treatment of asymptomatic, pauci-symptomatic and mild COVID-19 patients with lactoferrin (a milk-derived 80-kDa glycoprotein) revealed faster virus negativization and faster clinical recovery in comparison with untreated patients [9].

Probiotics are live microorganisms which can provide health benefits when consumed. Probiotics can activate barrier function [114]. Outside sleep-inducing effects, milk products including yogurt could increase natural killer cell properties and decrease the hazard of respiratory infections [115]. The daily intake of probiotics was suggested to be advantageous to human health by strengthening the immune reaction by regulating the gut bacterial ecosystem and enhancing the antiviral defense. Probiotics act with macrophages to promote the formation of interleukin-12 that activates the generation of interferon-γ, a principal antiviral cytokine. Moreover, yogurt bioactive peptides have powerful angiotensin-converting enzyme-inhibitory and bradykinin-enhancing properties. Hence, they can be efficient in the fight against the COVID-19 disease and its prejudicial health outcomes. Thus, it was demonstrated that the uptake of probiotics from milk significantly decreased the prevalence of respiratory tract contamination [7]. Prevention of respiratory syncytial virus infection with probiotic lactic acid bacterium *Lactobacillus gasseri* was determined in experimental models by Eguchi et al. [118].

COVID-19 individuals can exhibit intestinal microbial dysbiosis described by minimal numbers of different probiotic species such as *Bifidobacterium* and *Lactobacillus*, thereby presumably needing probiotic consumption to re-establish the intestinal flora equilibrium and reduce the hazards linked to COVID-19 [119]. A dietary source of probiotics is fermented foods and numerous research studies explore the efficiency of probiotics in treating or preventing COVID-19. Chourasia et al. [120] investigated the ability of 1420 bioactive peptides characterized in soy cheese peptidome developed by *Lactobacillus delbrueckii* in the inhibition of the SARS-CoV-2 main protease and S1 glycoprotein. Furthermore, a computer-guided research study determined the capacity of two bioactive peptides produced by β-lactoglobulin from goat milk whey fraction in inhibiting SARS-CoV-2 and angiotensin-converting enzyme [121].

### 4.2. Micronutrients (Vitamins and Minerals)

Micronutrients are gaining considerable attentiveness worldwide over the COVID-19 pandemic for their capability to affect the sensitivity to infection. Immunity implies vitamins renewing the ability of cells to produce some cytokines that impact the processes of immune cells. Among the cited vitamins, such as vitamin B complexes and vitamin C (water-soluble vitamins), vitamin A, vitamin D and vitamin E, vitamin E is a fat-soluble vitamin which covers a set of eight organic molecules, four tocopherols (α-, β-, γ-, and δ-tocopherol) and four tocotrienols (α-, β-, γ-, and δ-tocotrienol) [122]. These vitamins combined with some trace elements such as iron and magnesium were employed across the world to treat individuals affected by COVID-19 [1].

#### 4.2.1. Water-Soluble Vitamins

Vitamin B-complexes have different functions in the treatment of COVID-19. Neutrophil infiltration into the lungs could be highly prevented by vitamin B3 usage which showed anti-inflammatory properties during ventilator-caused lung injury [123]. It is found in numerous foods such as brown rice, legumes, sunflower seeds and nuts, bananas, citrus fruits, dark leafy vegetables, red meat, poultry, fish, eggs, salmon, liver and other organ meats, milk, cheese, oysters, mussels, pork, cheese, yogurt, nutritional and brewer’s yeasts and fortified cereal [1].

Vitamin B6 can help in the treatment of COVID-19 patients since they are subject to a lack of oxygen. Deficit in vitamin B6 will immediately inhibit the hemoglobin biosynthesis guiding the diminution of oxygen content in the body. In grave instances, it is among the principal causes of deaths. Diets with abundant vitamin B6 may be a substitute resolution. The foods rich in this vitamin are bread, whole grain cereals (brown rice, oatmeal), vegetables, soybean, potatoes, banana, spinach, seeds, carrot, pork, fish, poultry (chicken, turkey), eggs, milk and beef liver [1].

Vitamin C (ascorbic acid), a powerful antioxidant, neutralizes free radicals and assists in the prevention or reversal of cellular damage and it also has immunomodulatory effect [34]. Vitamin C possesses a role in the epithelial and endothelial barrier function, sustains vasodilatation and diminishes pro-inflammatory modulators [124]. Vitamin C has vital roles in the improvement of phagocytosis, chemotaxis and production of ROS, reducing necrosis and tissue injury [125]. It also plays a substantial function in the immune system, as since this vitamin practices an antioxidant effect by offering electrons, it defends cells against oxidative stress generated by infections, particularly infections impacting the lungs. Vitamin C enhanced functionality of phagocytes, proliferation of T-lymphocytes and formation of interferon, even lowering the replication of viruses. In the case of the COVID-19 pandemic, as 167 patients in the USA were provided 15 mg/day IV of vitamin C, a decline in the death rate was noticed [126]. 

Vitamin C was employed in 2003 during the SARS-CoV-1 emergence and as a nonspecific therapy for various respiratory tract infections [127]. Administering vitamin C to patients is an efficacious and economic scheme for the COVID-19 pandemic. The amounts of vitamin C in the serum of severely affected persons by COVID-19 found in the intensive care units were undetected or very bass [128]. Administering vitamin C shortened the patient stay in intensive care units by 8.6% and the period under mechanical ventilation by 18.2% [129,130]. 

Vitamin C is abundant in citrus fruits and red peppers, and the sources of vitamin C also include mangoes, broccoli, cauliflower, sweet potato, strawberries, tomatoes, papaya, beef liver, oysters, liver and eggs [1,115]. 

#### 4.2.2. Fat-Soluble Vitamins: Vitamin D and Vitamin E

Evidence of a reduction in vitamin D in the serum of COVID-19 patients has been reported since the early stages of the pandemic [131]. 

Vitamin D diminishes the hazard of usual colds and infections including COVID-19 via three processes namely a physical barrier, cellular natural immunity, adaptive immunity and stimulates the liberation of antimicrobial peptides [127]. Pro-inflammatory cytokines and B cells were down regulated, and anti-inflammatory cytokines were up regulated by vitamin D in the adaptive immune system [132]. The occurrence of the vitamin D receptor in most immune cells proposes its crucial role in immune cell properties [133]. Vitamin D deficit in winter is linked to viral epidemics. Moreover, vitamin D preserves respiratory tract protecting tight junctions, killing enveloped viruses through the induction of cathelicidin and defensins, declining the formation of pro-inflammatory cytokines by the innate immune system and diminishing the hazard of a cytokine storm which could lead to pneumonia [115]. 

The incidence of vitamin D deficit in ill persons with intense COVID-19 symptoms found in intensive care units was elevated (96.82%), in comparison with patients without any manifestations (32.96%) [134]. A retrospective work performed in the Philippines on 212 persons revealed an essential relationship between the vitamin D level in the plasma of COVID-19 patients and clinical outcomes [135]. 

Severe COVID-19 patients displayed vitamin D deficits with lower serum levels of 25-hydroxyvitamin D and with higher levels of inflammatory markers compared to asymptomatic patients. Moreover, low levels of 25-hydroxyvitamin are related to the severity of COVID-19. Patients treated with a high dose of cholecalciferol addition exhibited higher SARS-CoV-2 negativization than those who do not have the addition. Furthermore, the initial usage of vitamin D is connected with augmented survival between COVID-19 hospitalized patients. An adequate vitamin D status diminishes the employment of intensive care and results in a diminution in death. The initial uptake of an elevated dose of vitamin D in association with hydroxychloroquine and azithromycin significantly decreases the disease severity and access to intensive care compared to treatment with hydroxychloroquine or azithromycin alone [9]. 

It is important to note that eating a diet rich in vitamin D may help prevent infection with COVID-19. Furthermore, because the time spent outside and therefore sun exposure is restricted, it is recommended that individuals receive more vitamin D from foods. Foods that include vitamin D are fish, seaweeds, oat, soy milk, cereal, marine fish, beef liver, cheese, egg yolk, milk, shrimp, mushrooms, cheese, fortified soy milk, fortified cereal and foods with added vitamin D (e.g., milk, yogurt) [1,115].

Vitamin E, which covers a set of eight organic molecules, four tocopherols (α-, β-, γ-, and δ-tocopherol) and four tocotrienols (α-, β-, γ-, and δ-tocotrienol) [122], can exert multiple various immunological properties and can act as antioxidant, inhibiting the activity of protein kinase C and eventually interacting with transport proteins and enzymes [136]. Vitamin E is indispensable to get rid of chronic viral infections and decrease the rate of inflammation. Thus, vitamin E must be used in a sufficient content to diminish the probability of being infected by SARS-CoV-2. 

Vitamin E supplementation at 500 mg/kg may act as a treatment drug to inhibit ferroptosis in COVID-19 patients and decline ferroptosis damages to multiple organs, namely the lungs, kidneys, liver, gut, heart and nervous system. It reduces the ferric iron center in 15-lipoxygenase to inactive ferrous (Fe^2+^), resulting in 15-lipoxygenase inhibition and preventing lipid hydroperoxides formation [137].

Foods rich in this vitamin are vegetable oils (soybean, sunflower, corn, wheat germ, and walnut), nuts, seeds, green leafy vegetables (spinach, and broccoli), marine fish, octopus, goose meat and vitamin E fortified oil [1,115].

### 4.3. Trace Elements

Various trace elements (metals: zinc, iron, copper; oligoelements: magnesium, selenium) have been revealed to display a satisfying impact on reinforcing the human immune response [1]. 

#### 4.3.1. Magnesium

Magnesium exerts different functions in the immune system and it is involved in both innate and acquired responses. It acts as a cofactor for participation in immunoglobulin biosynthesis and antibody generation [138]. Magnesium associated with vitamins B12 and D reduces patient demands for oxygen support and intensive care support in China [139]. Magnesium can be found in green leafy vegetables, banana, avocado, nuts, seeds, legumes, peas, spinach, oatmeal, seafood (Salmon, mackerel, tuna), shrimp, egg, milk, beef and chicken [1]. 

#### 4.3.2. Iron

Iron has a key function in systemic oxygen transfer and serves as an electron donor or acceptor in several biological properties [140]. SARS-CoV-2 intrudes the heme metabolism in the body by attacking the 1-β chain of hemoglobin and hence capturing porphyrin, which results in an iron deficit. This is responsible for the evolvement of regular acute respiratory tract infections. A suitable level of iron can protect from the respiratory tract infections in gravely infected coronavirus patients. People who are iron deficient can get iron by eating these foods: pumpkin seeds, nuts, oats, brown rice, spinach, beans, potatoes, organ meats, beef, spleen, clams, egg yolk, shrimp and dark chocolate [1]. However, during an intensified inflammatory state, cytokines, such as IL-6, stimulate ferritin and hepcidin synthesis which is the key iron regulatory substance [140].

#### 4.3.3. Zinc

Another vital trace element that is critical for the sustenance of the immune system is zinc. It prevented grave acute respiratory syndrome coronavirus RNA-dependent RNA polymerase template linking and extension in Vero-E6 cells [115]. Zinc inhibits SARS-CoV and retrovirus RNA polymerase activity [34]. 

Zinc and vitamin A are fundamental for a successful proliferative response for the immune system since they regularize cell division and act as a cofactor with both catalytic and structural roles in several proteins [114].

It is found that zinc can be employed to treat COVID-19 patients owing to its modulation of immune response and antiviral properties. Despite the fact that this element is most abundant in oysters, other common aliments to obtain zinc are constituted from poultry, red meat, nuts, pumpkin seeds, sesame seeds, beans, lentils, soybeans, whole grains, shellfish, dairy products, eggs, cheese, dark chocolate and cocoa powder [1,115].

#### 4.3.4. Selenium

Selenium is essential for optimum immune functioning and it is the selenoproteins that regulate immunity. Selenium deficit may result in immune incompetence which heightens the vulnerability to infections. The evidence for the relevance of the selenium level in infectious diseases including human immunodeficiency virus infection and chronic hepatitis C virus prevalence was demonstrated [141].

There is no reliable research realized on selenium to ascertain its effect on SARS-CoV-2. However, the results of Moghaddam et al. [142] consolidate the concept of a pertinent function of selenium for COVID-19 convalescence and promote the discussion on adjuvant selenium complementation in gravely ill and selenium-deficient patients.

Selenium complementation is immuno-stimulatory, and is determined by T cell proliferation, NK cell activity, innate immune cell functions and others [141].

Almonds, pumpkin seeds, sunflower seeds, whole, wheat bread, fish, eggs pork, beef, chicken and turkey contain considerable amounts of this trace element [1].

### 4.4. Polyunsaturated Fatty Acids

Fatty acids are part of the constitution of the lipid double layer of biological membranes. They perform several functions (energy source, signaling molecules and precursors for the synthesis of eicosanoids) and are related to perform different functions in immune cells. Essential fatty acids such as omega-3 fatty acids regulate immune properties through its effect on inflammatory response [1]. These fatty acids must be procured from foods of marine origin (fish, shrimp and oysters) or vegetables such as walnuts, canola oil, spinach and soybeans [1,143].

For the cardiovascular apparatus, n-3 polyunsaturated fatty acids improve non-controlled inflammatory reactions, decreasing oxidative stress and attenuating coagulopathy. The anti-inflammatory effects of these fatty acids perform a critical function in mitigating the uncontrollable immune reaction in the lungs resulting from viral infections that might be beneficial in the regulation of COVID-19 [143].

So, severely diseased patients undergoing intravenous diet therapy fortified with fish oil lipid emulsion had a diminished hazard for infection by 40% and sepsis by 56% and a decrease in hospital and intensive care unit sojourn by approximately two days [144]. In the same trend, in order to cure gravely ill patients with COVID-19 by inhibiting cytokine excretion and reducing the inflammatory reaction, a parenteral complementation with fish oil emulsions, including considerable levels of eicosapentaenoic acid and docosahexaenoic acid (DHA; C22:6 n − 3) (4–6 g/d), was adopted [145].

In a pilot investigation, blood omega-3 amount from 100 COVID-19 patients was conversely linked to the risk of mortality [9].

In a survey consisting of 240 subjects with COVID-19, where the first cluster experienced standard care and the second cluster received 2 g/day of eicosapentaenoic acid capsules, the effectiveness of this fatty acid in the treatment of the sickness was demonstrated [143].

Nutrient effect results demonstrated in relevant studies are summarized in Table 2.

**Figure 2:**
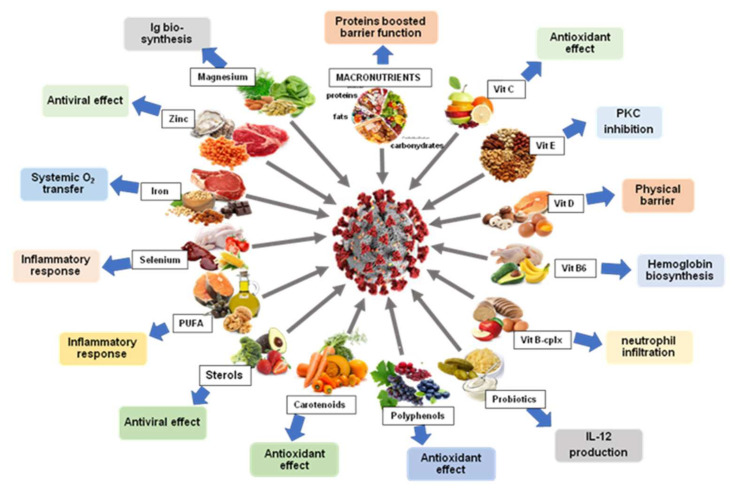
Different food classes with positive effects in the treatment of the COVID-19 infection.

A summary diagram of the main effects of different natural products (nutrients and secondary metabolites) is shown in Figure 2.

Nutritional recommendations and several food groups have been shown to be effective either in boosting up adaptive immunity or by acting as antivirals, anti-inflammatory and exogenous antioxidants. Among these food groups, we distinguish macro- and micronutrients (vitamins and minerals), trace elements (iron, zinc, magnesium, selenium), polyunsaturated fatty acids, different classes of secondary metabolites mainly phenolic compounds and terpenoids without forgetting other elements such as probiotics.

## 5. Perspectives and Emerging Technologies: Plant-Based SARS-CoV-2 Vaccines

Vaccination is currently the most efficient approach to prevent the proliferation of any viral illness [151,152]. The majority of the efficacious vaccine fabrication is performed by animal cell culture procedures. However, this takes several months to generate an important quantity of clinical grade vaccine doses. Other disadvantages are related to this method such as preservation, stabilization, recurrent contamination or infection of the cell culture systems [153].

Otherwise, the authorized vaccines were mostly founded on three common techniques of their production, (i) inactivated viral particles; (ii) mRNA formulated vaccines, and (iii) adenoviral or recombinant adenoviral vaccine [151]. Nevertheless, the usage of attenuated viruses and viral vectors in humans as vaccines may present some health hazards including the probability of mutation when using attenuated viruses and recombination by adopting viral vectors. Furthermore, the production of monoclonal antibodies towards COVID-19 cannot be a durable resolution because of prospective detrimental response [154].

Plant-based engineering, exploited in conventional and contemporary medication for several ailments such as infectious pathologies, possesses the ability to process efficacious, steady and cost-effective vaccines [155]. So, medicinal plant species may offer a resolution as an origin of natural antiviral constituents by the assemblage of secondary metabolites and also by performing as a platform to express the viral immunogenic proteins [156]. Secondary metabolites have an important function in protection due to biological properties and molecular farming is adopted for their large-scale synthesis. Moreover, metabolic technology can be employed to oppress the bioactive substance accessibility restrictions from herbal medicines and to enhance the productiveness advantages from bioprocessing and molecular farming. This consists of producing advisable recombinant proteins by whole plants or in vitro cultured plant tissues/cells. This can involve the additional refining of plant extracts, especially ones which have been employed formerly to satisfactorily inactivate SARS-CoV, as they can also have a role in inhibiting COVID-19 [154].

This is practicable to generate numerous sorts of vaccine, including protein subunit, virus-like particle (VLP), chimeric VLP and multiepitope vaccines, with the involvement of transient and stable expression. The plant transient expression process is quicker than any other procedure and offers a vaccine in 20 days after the protein amino acid sequence becomes accessible [157]. VLPs are made up of the plant lipid membrane as well as the COVID-19 spike protein, and they are comparable in dimension and form to the existent coronavirus; however, they do not contain nucleic acid so they are infective [154].

This methodology is extensively utilized for the manufacturing of recombinant proteins in various plant species including *Lactuca sativa*, *Arabidopsis thaliana*, *Nicotiana tabacum* and *Nicotiana benthamiana* which is the more suitable one [158]. The exploitation of tobacco plants for the expression of the coronavirus viral antigens can be a target for the prospect vaccinal investigation of the novel coronavirus because of the effectiveness of expression and intrinsic antiviral activities [156].

The traditional method for expression of transgenes in plants includes transgene inclusion into the nuclear genome. Presently, Agrobacterium-mediated transformation is the best popular procedure to attain this change. This bacterium possesses the capacity to transmit big stands of DNA with limited reorganization at elevated effectiveness with a bass number of insertions [152].

Vaccines produced in plants have been reeled to evoke a powerful immune reaction in humans [154]. These vaccines are perceived as third-generation vaccines [155], are secure, do not contain any consequent human pathogenic infection and are mainly high-quality clinical grade doses which can be developed within weeks. Distribution is the key to all vaccination efforts, particularly in developing countries [152].

Initial plant-based vaccines were produced for the Hepatitis B virus in 1992 [159]. Plant-based vaccines for COVID-19 can be generated in two ways: (1) By expressing the antigenic constituent of SARS-CoV-2 to provoke active immunity or (2) by expressing the antibody towards the virus to give passive safeguarding [155]. Vaccines that use individual proteins as antigens in a prime boost system alongside an adequate adjuvant, or as VLPs with various viral antigens could be valuable against COVID-19 [156].

In the USA, professionals at Kentucky Bio-Processing (KBP) cloned a portion of the genetic sequence of SARS-CoV-2, which they employed to perform a prospective Ag which was included in *Nicotiana benthamiana* for fabrication. The vaccine has induced a positive immune reaction in pre-clinical assays and will be entering phase 1 human clinical assays [160]. In Thailand, the aiyaPharming™ protein expression platform was used to produce a subunit-based vaccine to target COVID-19, also in *N. benthamiana* [158]. In South Africa, Cape Bio Pharms (CBP) is making spike S1 reagents composed of different zones of the glycoprotein associated with several fusion proteins and is trying to fabricate antibodies against these proteins [154].

Interestingly, the stage I clinical assay study of a plant-based COVID-19 vaccine has been developed by MedicagoInc, Canada. In this technique, *Nicotiana benthamiana* was used as plant system for the vaccine production and *Agrobacterium tumefaciens* as bacterial vector which is found in soils and is not toxic to plants. It has the ability to transfer its genetic material into the plant cells. This bacterium is injected with a plasmid containing the gene that codes for the spike protein of the coronavirus [161].

Potential targets included the spike, nucleocapsid, membrane, envelope, viral RNA polymerase and 3-chymotrypsin-like protease (3CLpro), that splits the virus polyprotein at 11 different positions to produce numerous non-structural proteins which are primordial for viral replication, all these proteins may be utilized to formulate eventual vaccines [154]. The main target being the S protein and its antigenic identification revealed central immunogenic proteins that can be expressed in the plants for the fabrication of a plant-based vaccine towards COVID-19. Thus, antibodies developed against the receptor-binding domain (RBD) of the S protein have been demonstrated to counteract COVID-19 [156].

However, it should be noted that various novel variants of COVID-19 are appearing worldwide, and existing vaccines are less sensitive to them. The cause can be the elevated proportions of S protein mutations, since the majority of the vaccines are focused on this protein. Therefore, new vaccines targeting other proteins, such as the N protein, can be considered a good alternative. The N protein is greatly immunogenic and highly preserved, offering lower susceptibility to mutation. Moreover, immunization by several antigens can offer satisfying preservation, suppressing the necessity for an amplifier dose. In line with this, the production of multiepitope and multivalent vaccines incorporated with plants might be effectual procedures to resolving the difficulties generated by COVID-19 mutational variants [157].

## 6. Conclusions

Several researchers have carried out state of the art in order to improve the understanding of the emergence of the new COVID-19 pandemic. Some of them have focused on medicinal plants and on their bioactive substances. We highlight the importance of natural products in COVID-19 pathogenesis complications and draw attention towards medicinal plants and different foods’ potential roles in mitigating the pandemic complications. Currently, there is direct evidence of a beneficial impact of numerous biologically active metabolites in COVID-19 patients. Indeed, biologically active molecules found either in medicinal plants or in diet possess the ability to regulate several of the detrimental actions caused by infection. Depending on the nature of the compounds, they are involved in different processes: they can modulate an excessive immune response, inactivate enveloped viruses, enhance macrophage phagocytic capability, improve coagulopathy, amend cell signaling and gene expression, change the model of the lipid metabolites generated under stress conditions to a more anti-inflammatory metabolite profile and activate the antioxidative property of the body.

Furthermore, numerous food ingredients determine the gut microbial composition and can consequently contribute to better regulate the immune response. A diet combined with medicinal plants with immunomodulatory, antiviral and anti-inflammatory properties can highly activate this safeguard. Diet must be abundant in vitamins and antioxidants, such as vitamins C, D, E, zinc, selenium and polyunsaturated fatty acids which have immunomodulatory activities and are substantial in protecting against the COVID-19 infection.

Additional studies, haphazard controlling assays and observational surveys are however needed to check and convert these advocated properties in the prevention and/or treatment of the actual COVID-19 infection and other possible infections. In addition, it should be noted that despite medicinal plants can have great potential against COVID-19, some of their compounds can also be toxic, teratogenic, mutagenic and carcinogenic. Hence, toxicological and pharmacological research studies are required to determine the safety and efficiency profile of the considered preparations and products. Herbal self-medication for serious diseases such as COVID-19 infection should be used with caution. However, as several foods and herbs possess immunomodulatory, anti-inflammatory and antiviral activities, and as a balanced diet and dietary intake of nutrients affect the immune system in many ways, the use of natural molecules from plants is an attractive nutritional and pharmacological approach that should not be overlooked and deserves to be explored in order to prevent and deal with pandemics, the scale of which may have significant and detrimental socioeconomic consequences.

All in all, extensive randomized, controlled investigations are required to appoint the functions of medicinal plants and of their active molecules, micro- and/or macronutrients in the various phases of COVID-19 and to assay their favorable and/or negative impacts, before the endorsement of their therapeutic usage in infected patients.

## Figures and Tables

**Table 1:** Medicinal plants efficient against COVID-19 infection.

Latin/Vernacular Names	Nature of Extracts or Components	Mode of Action	Refs.
*Aegle marmelos* L./Bael	Purified seselin	Inhibitory potential over multiple SARS-COV-2 targets such as SARS-CoV-2S spike protein, COVID-19 main protease and free enzyme of the SARS-CoV-2 (2019-nCoV) main protease.	[103]
*Anacyclus pyrethrum* L./Akarkara	Pyrethrin	Acts as ligands with viral proteins to prevent the binding of host receptors and the fusion leading to viral replication.	[104]
*Andrographis paniculata* Burm.f./Creat	Andrographolide/Andrographiside	ACE inhibition/SARS-3CLpro inhibition of the NOD-like receptor protein 3 (NLRP3), caspase-1 and interleukin-1β (IL-1β) particules.	[11,39,105]
*Asparagus racemosus* L./Willd	Hydroalcoholic crude extract	ACE inhibition/IC_50_ = 82.88%.	[106]
*Camellia sinensis* L./Tea plant	Polyphenol (Rutin), theaflavin-3,30-digallate, tannic acid, [−]-epigallocatechin gallate)	ACE inhibition, SARS-3CLpro inhibition.	[105]
*Carapichea ipecacuanha* L./Ipecacuanha	Emetine	Displayed strong anti-CoV activity by blocking MERSCoV entry consistent with pseudovirus entry assays.	[107]
*Citrus* Spp./Citrus	Hesperetin, hesperidin Rhoifolin, Neohesperidin	SARS-3CLpro inhibition in a dose-dependent manner.	[105]
*Curcuma longa* L./Turmeric	Curcumin and its analogue	In a molecular docking study, curcumin and few of its derivatives are suggested as SARS-CoV-2 spike protein inhibitors.	[11]
*Cynara scolymus* L./Globe artichoke	Cynaroside	ACE inhibition/IC_50_ = 49.7%	[105]
*Dioscorea batatas* L./Chinese yam	-	SARS-3CLpro inhibition/ IC_50_ = 44 μg/mL.	[105]
*Erigeron abajoensis* L./Cronquist	Flavone (Scutellarin)	ACE inhibition.	[105]
*Equisetum hyemale* L./Rough horsetail	Herbacetin	3CL inhibitory activity.	[105]
*Galla chinensis* L./Chinese sumac	Tetra-*O*-galloyl-β-D-glucose	Binding with surface spike protein of SARS-CoV.	[108]
*Glycyrrhiza glabra* L./Black sugar	Glycyrrhizin	Inhibition of COVID-19 replication and entry to its host cells. Glycyrrhizin can inhibit ACE with IC_50_ > 40%.	[105,108,109]
*Linum usitatissimum* L./Linseed	Herbacetin	SARS-3CLpro inhibition.	[105]
*Hancornia speciosa* L./Gomes	Chlorogenic acid	ACE inhibition.	[105]
*Houttuynia cordata* Thunb./Fish mint	The aqueous extract	Inhibition of RNA-dependent RNA polymerase (RdRp), 3CL-like protease and viral polymerase.	[108,109,110,111]
*Hypericum perforatum* L./St. Johnswort	Hypericin	C-terminal and N-terminal domains of 2019-nCoV NSP 14 can bind Hypericin.	[108]
*Isatis indigotica* L./Woad	Phenol (indigo, sinigrin, aloe emodin, hesperetin, sinigrin), 2,2-Di(3-indolyl)-3-indolone, phaitanthrin D	Inhibit the cleavage activity of SARS-3CLpro enzyme/IC_50_ = 53.8 ± 4.2 μg/mL.	[105,108,109]
*Litchi chinensis* L./Litchee	Flavonoids such as rhoifolin, pectolinarin, Epigallocatechin gallate, Gallocatechin gallate, quercetin and herbacetin	Inhibition of SARS-3CLpro activity.	[109]
*Lycoris radiata* L./Red spider lily	Glycyrrhizic acid derivatives	Reduction or inhibition of penetration and viral attachment (IC_50_ = 2.4 ± 0.2 μg/mL).	[110]
*Nigella sativa* L./Black cumin	Nigellidine and α-hederin	High potential to act as COVID-19 treatment in docking studies.	[50]
*Ocimum sanctum* L./Holy basil	Tulsinol and dihydroeugenol	Effective against SARS CoV 2 in molecular docking studies.	[11]
*Polygonum Multiflorum* Thunb./Chinese knotweed	Emodin	Inhibit interaction of SARS-CoV spike protein and ACE2. Inhibit the 3a ion channel of coronavirus SARS-CoV.	[108,111]
*Psoralea corylifolia* L./Purple fleabane	Bavachinin, psoralidin Corylifol	The ethanol extracts of these secondary metabolites show high activity against SARS-CoVPLpro.	[111]
*Rheum officinale* Baill./Chinese rhubarb	Anthraquinone (Emodin)	Positive ACE inhibitor in combination with ACEI/ARB agents. Inhibition of the interaction between SARS-CoV S (IC_50_ = 1 to 10 μg/mL). In a dose-dependent manner, it drastically blocked the interaction of the ACE2 enzyme of host cell and viral S protein.	[105,110,111]
*Sambucus Formosana Nakai*/Chinese elder	Ethanol extract	Significant reduction in virus yield, plaque formation and virus attachment.	[36]
*Scutellaria baicalensis* L./Georgi	Baicalin, cosmosiin	ACE inhibition and SARS-3CLpro inhibition.	[105,108,109]
*Toona sinensis* Roem./Chinese mahogany	Quercetin and TSL-1	Inhibition of the cellular entry of SARS-CoV.	[108]
*Torreya nucifera* L./Japanese torreya	Amentoflavone and Apigenin	Showed the most potent 3CLpro inhibitory effect.	[111]
*Tylophora indica* L./Indian ipecac	Tylophorine	Tylophorine-based biomolecules exhibit broad spectrum potential for inhibiting coronaviruses.	[108]
*Veronicalina riifolia* L./Speedwell	Luteolin	Avidly binds with surface spike protein of SARS-CoV.	[108]
*Withania somnifera* (L.) Dunal/Winter cherry	Withanone and withaferin	Effective against SARS CoV-2 in bioinformatic studies. In molecular docking, inhibitors against SARS-CoV-2 Mpro (Main protease).	[11,112]

**Table 2:** Mode of action of some nutrients tested against COVID-19.

Nutrients Types	Mode of Action against COVID-19	Refs.
Macronutrients		
Protein	Oral and IV glutathione, glutathione precursors (N-acetyl-cysteine) block NF-κB. A trial of 2 g of IV improved dyspnea of patients within 1 h of use. Repeated use of both 2000 mg IV glutathione was effective in further relieving respiratory symptoms.	[126]
Polyunsaturated fatty acids	Suppress inflammation and augment phagocytosis. Exhibit anti-inflammatory, vasodilatory and platelet anti-aggregatory effects.	[146]
Probiotics	Inhibit SARS-CoV-2 main protease, S1 glycoprotein and angiotensin-converting enzyme.	[120,121]
Micronutrients		
Vitamin B-complexes	Vit B1 acts as a carbonic anhydrase isoenzyme inhibitor. Vit B2-UV decreases the infectious titer of SARS-CoV-2 below the limit of detection in human blood and in plasma and platelet products. Vit B9 and its derivatives have strong and stable binding affinities against the SARS-CoV-2, through structure-based molecular docking.	[147]
Vitamin C	Inhibits cytokine storm through reducing inflammation rate and respiratory tract infection	[1]
Vitamin D	Vitamin D tablets can be taken to reduce mortality rate and suppress cytokine storm in the human body.	[1]
Vitamin E	Inactivation of 15-lipoxygenase by the reduction of Fe^3+^ to Fe^2+^ leading to ferroptosis prevention.	[137]
Magnesium	Reduction in lung inflammation response and oxidative stress, and inhibition of bronchial smooth muscle contraction; favors bronchodilation.	[148]
Zinc	Doxycycline, a tetracycline antibiotic, is known to chelate Zn from matrix metalloproteinases, which may help in part to inhibit the COVID-19 infection by limiting its ability to replicate in the host.	[149]
Selenium	Enhance adaptive immunity by reinvigorating cytotoxic cells and moderating the release of inflammatory cytokines by the innate immune system.	[150]
B_12_ supplements (500 μg), vitamin D (1000 IU) and magnesium	Reduce COVID-19 symptom severity and the need for oxygen and intensive care support.	[147]
Vitamin C and E	Ameliorate cardiac injuries of critically ill COVID-19 patients.	[143]
Copper, Iodine, Selenium, Zinc	Immune enhancers towards SARS CoV 2.	[1]

## Data Availability

Not applicable.
